# Prevalence of periapical lesions in non-endodontically and endodontically treated teeth in an urban Iraqi adult subpopulation: A retrospective CBCT analysis

**DOI:** 10.4317/jced.59877

**Published:** 2022-11-01

**Authors:** Ahmed H. Ali, Anas F. Mahdee, Noor H. Fadhil, Duaa M. Shihab

**Affiliations:** 1 BDS, MSc and PhD (UK), Aesthetic and Restorative Dentistry Department, College of Dentistry, University of Baghdad, Baghdad, Iraq; 2 BDS and MSc, Aesthetic and Restorative Dentistry Department, College of Dentistry, University of Baghdad, Baghdad, Iraq; 3 BDS, Aesthetic and Restorative Dentistry Department, College of Dentistry, University of Baghdad, Baghdad, Iraq

## Abstract

**Background:**

This study aimed to assess the prevalence of apical periodontitis (AP) and its association with the presence/quality of root canal filling (RCF) and coronal restoration (CR) in Iraqi population.

**Material and Methods:**

A total of 385 CBCT scans of patients (18-45) yrs. old with 9250 teeth were examined. The teeth were grouped according to the presence/absence of apical radiolucency, presence/radiographic quality of RCF, and CR. Chi-square and Kappa were used to assess associations and intra-consensus reliability. Logistic regression was used to predict risk factors associated with AP. The significant level was set at *p*<0.05.

**Results:**

AP was prevalent in 17.7 and 80.2% of teeth without RCT and with RCT (*p*<0.05), respectively. AP in root canal-treated teeth with missed canals (93.2%) was higher than that in root canal-treated teeth with no missing canal (78.3%) (*p*<0.05). AP in teeth with inadequate RCF (87%) was higher than that in teeth with adequate RCF (63%) (*P*<0.05). No difference in the prevalence of AP in teeth with adequate vs inadequate CR ((79.7%) vs (81%), respectively) (*p*>0.05). The presence of AP was significantly associated with inadequate RCF (vs adequate RCF) (OR=4.16, CI 95% 2.29-7.56, *P*<0.05), and was not associated with inadequate CR (vs adequate CR) (OR=-0.71, CI 95% 0.35-1.42, *P*>0.05). Intra-consensus reliability was (0.9) for AP and (0.82) for RCF and CR quality.

**Conclusions:**

AP was highly prevalent in teeth with previous root canal filling compared to non-treated teeth. AP was significantly associated with inadequacy of root canal filling but not with the inadequacy of coronal restoration.

** Key words:**Apical periodontitis, cone beam computed tomography, root canal treatment, endodontics.

## Introduction

The favorable outcome of root canal treatment has been reported to be high, however, in reality, only 35%-60% of root canal-treated teeth demonstrate normal apical periodontal condition in radiographic cross-sectional studies (1,2). Radiographic pathology associated with the apex of the root is usually referred to as apical periodontitis (AP), which is an inflammatory response to microbial infection of the root canal system pre/post root canal treatment (3). Many epidemiological studies assessed the prevalence of AP within different populations across the globe (4-6), The data of these studies represent the actual outcome of root canal treatments in the overall population and many of them found a significant association between periradicular health/pathosis and the presence/quality of root canal filling (RCF) (2,6-8). There were links between the presence of AP and systemic low-grade inflammation and health impairment (9,10). Also, assessing the prevalence of AP and risk factors in a population may help to predict the future need for the dental treatment of that population and also to monitor the performance of the dental profession.

This cross-sectional study aimed to assess the prevalence of AP in teeth of an adult Iraqi population and its association with the absence/presence of RCF, its radiographic quality, and coronal restoration adequacy.

## Material and Methods

This study was approved by the academic research ethics committee at the College of Dentistry/ University of Baghdad. The sample size was calculated to be 385 based on error margin of 5% and the Iraqi population is about 40 million people. A total of 385 full-size scans were randomly selected and obtained from five different devices (CARESTREAM 8100 3D, France, 2017) (Villa, Italy, 2017) (GALILIOS Sirona comfort PLUS unit, Germany) (GENDEX, GDDP-700-1, Finland, 2014) (WhiteFox control, Australia, 2017), and displayed by installed or compact disc built-in software (CS 3D imaging v 3.8.6) (dental studio) (GALILEOS viewer v1.9) (Invivo dental viewer v 5.1) (white fox imaging) correspondingly. All software had similar functionalities to allow for consistent assessment methodology. Technical settings and parameters were in the following range: FOV (8×9 to 16×17 mm2), voxel size (0.125 to 0.200 mm3), slice thickness (0.15 to 1 mm), exposure time (15 to 10.08 s), tube voltage (80 to 105 Kvp), and tube current (3.2 to 10 mA). These samples were gained from a pool of archived data from the dental radiographic departments of both governmental and private health institutions. They were for patients between 18-45 years old who had previously been referred from dental or maxillofacial clinics for reasons not related to this study, in the years from 2016 to 2021. Approval of the Research Ethics Committee of the College of Dentistry/ University of Baghdad has been acquired before commencing this retrospective radiographic study.

Images in axial, sagittal, and coronal sections of the target tooth were observed, analyzed, and documented by sliding the mouse pointer scrolling ring back and forth along each section. Magnification, contrast, and brightness were adjusted according to observer preference. The images were displayed on a 15.6-inch DESKTOP-NOBV05T laptop computer, with 1920×1080 screen resolution. Pre-calibration of an independent consensus panel, which consisted of two experienced endodontists in CBCT scans analysis, was performed using 30 CBCT scans that were not included in the study sample. They analyzed the samples to reach conformity, jointly. The intra-consensus agreement was assessed by repetition of the assessment of the CBCT scans together. Twenty-five examination was performed per day to avoid fatigue. In each scan, all maxillary and mandibular teeth were assessed according to the inclusion and exclusion criteria.

The assessment was performed at tooth level, all teeth were included in the assessment except for teeth undergone orthodontic treatments, retained roots, retained primary teeth, fractured root, pathologic lesions of non-dental origin, combined endo perio-lesions, impacted teeth and not fully erupted teeth. The teeth were grouped according to the presence/absence of apical radiolucency, which was defined as any lateral or apical radiolucency (that exceeds double the width of the adjacent normal periodontal ligament) associated with the apical part of the root. Also, teeth were grouped according to the presence/absence of RCF, and the teeth with RCF were categorized into adequate/inadequate RCF and adequate/inadequate coronal restoration according to the following criteria:

1- Endodontic treatment.

a- Adequate: All canals obturated with a homogenous root filling material with no voids present and the RCF ending not more than 2 mm short from the radiographic apex.

b- Inadequate: RCFs that are shorter than the radiographic apex by more than 2 mm or the filling material (including sealer) extend/pushed irregularly beyond the radiographic apex. RCF with, voids, unfilled canals, separated instruments, perforations, and ledges. In multirooted teeth, root with an apical radiolucency and inadequate RCF was considered within this group.

2- Coronal restoration:

a- Adequate: include intact permanent restoration and crowns.

b- Inadequate: include lost restoration, overhang, recurrent caries, open margin, and a temporary restoration.

Statistical analysis included Chi-square to assess associations of different variables and the prevalence of AP. Kappa was used to assess intra-consensus reliability. Logistic regression was used to predict risk factors associated with AP. The significant level was set at *p*<0.05. All calculations were performed using SPSS package version 26 (SPSS Inc., Chicago, IL, USA).

## Results

A total of 385 subjects/scans yielded 9250 teeth, the prevalence of AP among subjects and teeth in the study is shown in Table 1.

**Table 1:**
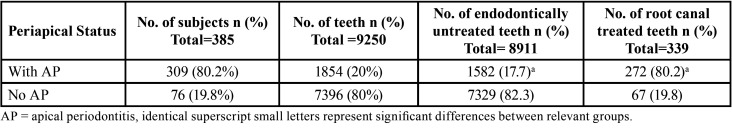
Prevalence of AP among subjects and teeth in the study.

The teeth without RCT and have AP distributed as follow 75 have an intact sound crown, 1434 teeth have caries or defective filling, 28 teeth have an intact filling and 45 teeth have crown restoration. The number of teeth with RCT was 339/9250 (4%) distributed over 73 subjects. Root canal-treated teeth in females were195 teeth and 144 teeth in males (57.5% vs 42.5%). The presence of AP was significantly higher in teeth with RCF than in untreated teeth (80.2% vs 17.7%) (*p*<0.05), as shown in Table 1.

No statistical difference in the prevalence of AP between females and males in the root canal-treated teeth (*p*>0.05). Root canal-treated teeth with missed canals were 44/339 (12.9%) of the total root canal-treated teeth, AP was prevalent in 41/44 (93.2%) of them. The prevalence of AP in root canal-treated teeth with missed canals (93.2%) was higher than that in the root canal-treated teeth with no missing canal (78.3%) (*p*<0.05). The prevalence of AP and missed canals in root canal-treated teeth according to the tooth type is shown in Table 2.

**Table 2:**
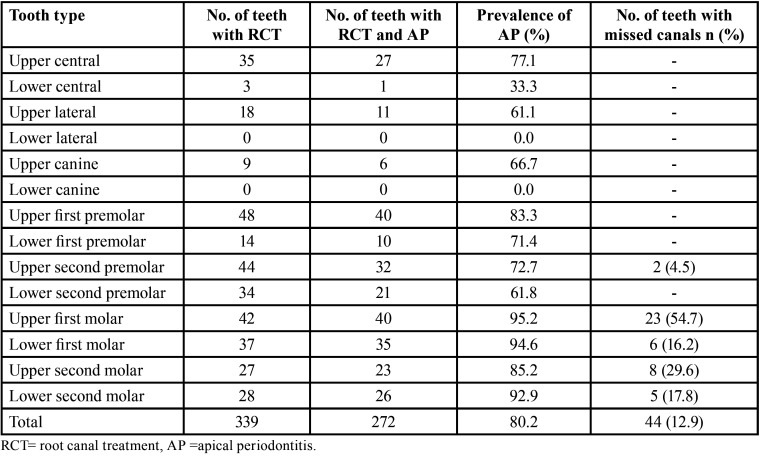
Prevalence of AP and missed canals in root canal treated teeth according to the tooth type.

Prevalence of AP in root canal-treated teeth according to the adequacy/inadequacy of RCF and coronal restoration is shown in Table 3. Inadequate RCFs were present in 247 teeth equal to 72.8% of the total root-filled teeth. In teeth with inadequate RCF, there was a higher prevalence of AP vs teeth with adequate RCF (*P*<0.05), however, there was no significant difference in the prevalence of AP in teeth with adequate vs inadequate coronal restoration (*p*>0.05), as shown in Table 3.

**Table 3:**
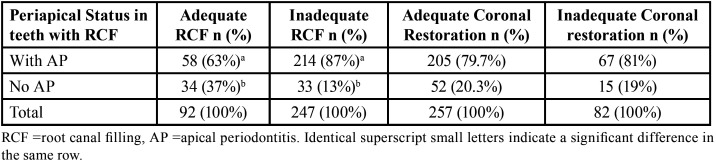
Prevalence of AP in endodontically treated teeth according to the adequacy/inadequacy of RCF and coronal restoration.

Risk factors associated with the prevalence of AP in root canal-treated teeth were assessed by conducting a binary logistic regression. The presence of AP was significantly associated with inadequate RCT (vs adequate RCT) (OR=4.16, CI 95% 2.29-7.56, *P*<0.05). On the other hand, the presence of AP was not associated with inadequate coronal restoration (vs adequate coronal restoration) (OR=-0.71, CI 95% 0.35-1.42, *P*>0.05), as shown in Table 4. Cohen Kappa for intra-consensus reliability was (0.9) for AP and (0.82) for RCT and coronal restoration quality, respectively.

**Table 4:**
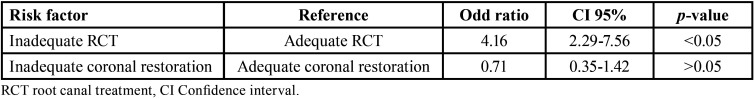
Binary logistic regression of risk factors of AP presence.

## Discussion

This cross-sectional study examined the prevalence of AP in a population of Iraqi adult individuals using CBCT for the first time with a total number of teeth equal to 9250 with an average of 24 teeth per subject. It is important to study the epidemiology of AP and the associated risk factors in the scope of linking between AP, systemic low-grade inflammation (10), and impairment of systemic health (9). Also, it may help to predict the future need for dental treatment of a population and also improve the outcomes of under and postgraduate education programs in the dental profession.

In this study, CBCT was utilized for assessing the prevalence of AP because of its higher diagnostic accuracy in the detection of apical radiolucency compared to dental panoramic and periapical radiographs (11). Although the diagnosis of true apical periodontitis can only be confirmed by histopathological examination, it is unethical to perform a biopsy in most cases. A recent report has shown the ability of CBCT to detect AP more accurately compared to periapical radiographs by using a histopathological reference on human *ex-vivo* specimens (12). In the present study, as in many other epidemiological studies, AP was defined as a radiolucency associated with a root apex that exceeds twice the width of normal adjacent periodontal ligament (13-15).

The prevalence of AP at the subject and tooth levels were higher in this study in comparison to the previous report (52% and 5%, respectively) (16). Many epidemiological studies reported a lower prevalence of AP compared to the results of this study (4,5,15,17), however, these reports utilized dental panoramic and dental periapical radiographs compared to this study which used CBCT. This could be the main cause for this higher prevalence of AP detection. Estrela *et al*. (2008), showed that AP was detected in 17.6%, 35.3%, and 63.3% of endodontically treated teeth using panoramic radiographs, periapical radiographs and CBCT, respectively (3). Two-dimensional radiographs tend to underestimate the prevalence of AP because lesions visualized by these techniques are required to have mineral bone loss reached about 30-50% (3). Therefore, CBCT can comprehensively detect AP lesions and this can help in quantifying the level of bone loss and thus improving the therapeutic decision and prognosis. However, over-diagnosis of AP using CBCT need to be considered (18). On the subject level, the results of this study are comparable to the results of other CBCT studies that assessed the prevalence of AP. The AP prevalence among subjects was 78% in a study by Lemagner *et al*. (2015) (19). Kabak and Abbott reported an 80% prevalence of AP on the subject level in a Belarusian population (14) in comparison to 80.2% for the current results.

At the tooth level, the overall prevalence of AP in all teeth in this study was 20% compared to 6.3%, 3.8%, and 10.4% in other studies (2,6,20) respectively. Although an average of 24 teeth per subject was recorded in this study, which indicates that patients tend to retain their teeth longer and avoid extractions and wearing dentures, the percentage of root-filled teeth was 4% in comparison to 12.2%, 20% and 8.2% in other studies (6,8,14), respectively. This could be attributed to the lower awareness of the study population toward endodontic treatment. Another cause for this difference could be the average age in this study was lower than that in the other mentioned studies which had been performed in European countries.

Although, the prevalence of AP in the untreated teeth in this study was 17.7% which is significantly lower than that for RCT teeth (80.2%). However, this is consistent with the results of other studies, that attempt to assess the risk factors for the radiographical detection of AP in different populations around the world. It was concluded that the presence of root filling in a tooth significantly increases the risk of detecting AP radiographically (6,8,14). Also, adequacy/inadequacy of the RCF significantly affected the prevalence of AP in this study. Inadequate root fillings were present in 72.8% of root-filled teeth in this study compared to 54.5% in root-filled teeth in a Belgian subpopulation (8), 81% in root-filled teeth in a French subpopulation (21), 55.8% in root-filled teeth in a Dutch subpopulation (22) and 50% in a Belarusian population (14). All above-mentioned studies in addition to the results of this study found significant correlations between the inadequacy of root filling and detecting AP in radiographs/CBCTs.

The percentage of root canal-treated teeth with missed canals, in this study, was 12% of the total number of root canal-treated teeth which is similar to that reported by Costa *et al*. (2018) and lower than that reported by Karabucak *et al*. (2016), which was 23% (23,24). Also, in this study, root canal-treated teeth with missed canals showed a higher prevalence of AP compared to that with no missing canals (93.2% vs 78.3%). This is similar to results obtained in previous studies, the results from Costa *et al*. (2018), showed that 98% of teeth with missed canals showed AP presence compared to only 86% of teeth with no missed canals (24). Another study by Karabucak *et al*. (2016) showed that a tooth with a missed canal was 4.38 times more likely to be associated with a lesion (23). The highest percentage of teeth with missed canals was in the upper first molars and upper second molars (54.7% and 29.6%, respectively) (mean= 44.9%). Similarly in other studies, upper molar teeth exhibited the highest proportion of root-filled teeth with missed canals 40.1% and 57% (23,24), respectively. This indicates the necessity to consider most of the time that the upper molars are four canals teeth and efforts are required to locate the mesio-buccal canals by the clinicians using magnifying tools such as; loupes and dental operating microscope which can significantly improve the detection rate of MB canals (25).

Prevalence of AP in endodontically treated teeth was 80.2% in this study which is higher than other studies that used CBCT scans (38.2%, 59.5%, 42.5%), respectively (6,19,23). This indicates the need to improve the quality of dental care, especially root canal treatment, provided to the patients to reverse the present state. This possibly can be performed through investing more in developing the training skills for general dental practitioners or maybe by limiting root canal treatment to be provided by endodontists only. Also, the situation needs to be evaluated periodically in the short and long term to ensure monitoring of the outcome effect of implementing such strategies. Otherwise, a continuous increase in the prevalence of apical periodontitis could be expected in the future (20).

One of the limitations of this study was the data collection which was not capturing all the causative factors for the outcome of RCT such as the time of the treatment, skill of the operator, treatment methods and materials. Although it is impossible to determine whether the detected AP is healing or expanding in epidemiological studies but it has been found that after 10 years, the number of newly developed lesions was equal to the healing lesions (26). Another limitation is that the CBCT scans of the study population were acquired from archived data and not from a random population sample, therefore results should be applied to the general population with caution, as including CBCTs of patients with an endodontic problem could have increased the prevalence of AP in this study population. However, it is unethical to perform a randomized trial and expose the population to radiation of CBCT to acquire the data.

## Conclusions

AP was significantly higher in root canal treated teeth compared to not treated ones and inadequate root canal filling significantly increase the odds of having AP compared to adequate root canal filling making it a risk factor for developing AP. On the other hand, coronal restoration adequacy is not associated with the risk of presence/absence of AP in root canal-treated teeth. Teeth with root canal filling and missed canals have a higher prevalence of AP compared to teeth with no missing canals.
